# Manual Segmentation of 12 Layers of the Retina and Choroid through SD-OCT in Intermediate AMD: Repeatability and Reproducibility

**DOI:** 10.18502/jovr.v16i3.9435

**Published:** 2021-07-29

**Authors:** Pedro Camacho, Marco Dutra-Medeiros, Luís Salgueiro, Sílvia Sadio, Paulo C. Rosa

**Affiliations:** ^1^H&TRC – Health & Technology Research Center, ESTeSL – Escola Superior de Tecnologia da Saúde, Instituto Politécnico de Lisboa, Lisbon, Portugal; ^2^Ophtalmology Institute Dr. Gama Pinto, Lisbon, Portugal; ^3^Central Lisbon Hospital Center, Lisbon, Portugal; ^4^Retina Institute of Lisbon, Lisbon, Portugal; ^5^NOVA Medical School, Lisbon, Portugal

**Keywords:** Data Accuracy, Diagnostic Imaging, Macular Degeneration

## Abstract

**Purpose:**

To evaluate the repeatability and reproducibility of the segmentation of 12 layers of the retina and the choroid, performed manually by SD-OCT, along the horizontal meridian at three different temporal moments, and to evaluate its concordance with the same measurements performed by two other operators in intermediate AMD.

**Methods:**

A cross-sectional study of 40 eyes from 40 subjects with intermediate AMD was conducted. The segmentation was performed manually, using SD-OCT. The 169 measurements per eye were repeated at three time points to study the intra-operator variability. The same process was repeated a single time by two different trained operators for the inter-operator variability.

**Results:**

Forty participants (28 women and 12 men) were enrolled in this study, with an average age of 76.4 ± 8.2 (range, 55–92 years). Overall, the maximum values of the various structures were found in the 3 mm of the macula. Intra-operator variability: the highest ICC values turned out to be discovered in thicker locations. Inter-operator variability: except correlation values of 0.826 (0.727; 0.898) obtained in the OPL (T2.5) and 0.634 (0.469; 0.771) obtained in the IPL (N2), all other correlation values were > 0.92, in most cases approaching higher values like 0.98.

**Conclusion:**

The measurements of several layers of the retina and the choroid achieved at 13 locations presented a good repeatability and reproducibility. Manual quantification is still an alternative for the weaknesses of automatic segmentation. Locations of greatest concordance should be those used for the clinical control and monitoring.

##  INTRODUCTION

Age-related macular degeneration (AMD), an ocular pathology resulting from the interaction between genetic components and environmental factors,^[1]^ is one of the leading causes of irreversible blindness.^[2]^

The development and growth of spectral-domain optical coherence tomography (SD-OCT) have revolutionized the field of ophthalmology.^[1,2,3]^ The AMD histological data retrieved by SD-OCT are crucial, and thickening of the ganglion cell complex^[4]^ or the photoreceptors, and atrophy or hyperpigmentation of the retinal pigment epithelium (RPE) have been described.[4–6] However, an accurate analysis of the different retinal layers is essential to differentiate the normal human retinal aging process from the progression of initial and intermediate forms to late-stage forms of AMD.^[4,5,6]^

Several studies involving healthy participants reported high correlation values for intra- and inter-operator variability, ensuring good consistency between different visits and observers.^[1,5,7,8,9]^ Unfortunately, the automatic segmentation in the presence of pathology still has some limitations.^[10]^ The evaluation of AMD features and associated changes in retinal layers has become crucial in AMD phenotyping.^[5,7,8,11,12]^ However, accurate layer segmentation, which is crucial for appropriate clinical interpretation, remains an interesting OCT research area. In addition, this still presents difficult challenges even in the deep learning (DL) approach due to the presence of structural changes in the retina.^[13,14]^

Despite the complexity and time consumption, especially in large studies,^[13,15]^ the quantification and variability of the segmentation of retinal layers need to be optimized and standardized.^[7]^ Currently, there are no studies on

the validation and evaluation of the repeatability and reproducibility of segmentation of 12 layers of the retina and the choroid performed manually by SD-OCT in intermediate AMD (iAMD).

##  METHODS

### Participants

All procedures in this study and data collection followed the principles of the Declaration of Helsinki. All subjects provided informed consent, and the study protocol was approved by the Ethics Committee of the Ophthalmology Institute Dr. Gama Pinto (IOGP).

Consecutive patients with iAMD (category 3 of the Age-Related Eye Disease Study [AREDS] classification), followed-up at the Retina Department of the IOGP between January 2014 and July 2015, were retrospectively recruited from an initial pool of patients classified as having early/intermediate AMD.^[4]^ In this study, we only included cases with digital color fundus photographs obtained and graded according to the AREDS classification system.^[16]^ All participants had best-corrected visual acuity (BCVA) obtained with the Early Treatment Diabetic Retinopathy Study chart.

### Exclusion criteria

Spherical equivalent refractive error greater than ± 6.0 diopters; the opacity of the media which prevented the correct visualization and quantification of retinal layers, off-center image, subfoveal hemorrhage, ocular inflammation, history of retinal detachment, retinal serous detachment, photodynamic therapy, or any other pathology (including advanced AMD), previous ocular surgery close to the region corresponding to the OCT data, history of ocular trauma in the studied eye; glaucoma (including the suspicion of the optic nerve and/or intraocular pressure [IOP] ≥ 19 mmHg), intravitreal injection (even of triamcinolone); a clinical history of stroke, transient ischemia, dementia, and/or other neurological disorders.

### Procedures

**Figure 1:**
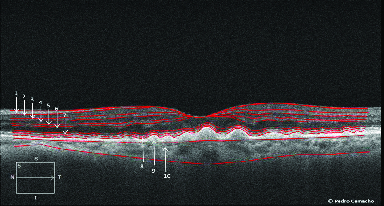
1, (RNFL) retinal nerve fiber layer; 2, (GCL) ganglion cell layer; 3, (IPL) inner plexiform layer; 2+3, (GCL+IPL) ganglion cell layer with inner plexiform layer; 4, (INL) inner nuclear layer; 5, (OPL) outer plexiform layer; 6, (ONL) outer nuclear layer; 5+6, (OPL+ONL) outer plexiform layer with outer nuclear layer 7, (MZ) myoid zone of the photoreceptors; 8, (OS) outer segments of photoreceptors; 9, RPE+BM complex (interdigitation between apical processes of the RPE and external portion of the external segments of the photoreceptors and Bruch membrane); 10, choroid thickness.

**Figure 2:**
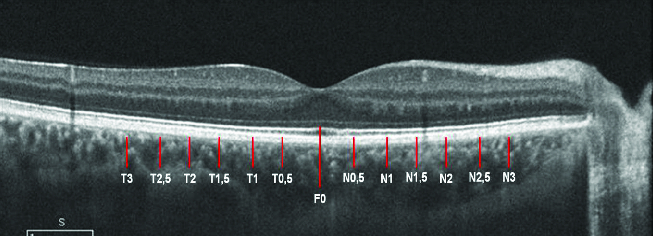
Representation of the 13 locations studied at intervals of 0.5 mm from the center of the fovea. T, temporal values; F0, foveal values; N, nasal values.

Using a manual approach, we included 40 eyes from 40 participants. From that sample, the retinal layers and the choroid [Figure 1] were segmented and quantified in the horizontal meridian [Figure 2], from the fovea (F0) at 13 locations. We repeated each measurement three times, with an interval of at least a week for intra-operator analysis. Additionally, two operators from the same institution and masked to the previous measurements repeated the measurements and the quantification of the 40 eyes. These data were also collected and compared to the key operator for the inter-operator study.

**Figure 3:**
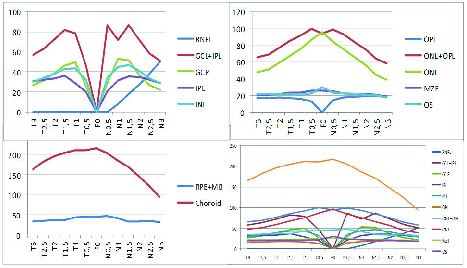
Representation of the 13 locations studied at intervals of approximately 0.5 mm from the center of the fovea. (A) Inner layers. (B) Outer layers. (C) RPE+BM complex and choroid. (D) Total layers in the study. T3, 3 mm temporal; T2.5, 2.5 mm temporal; T2, 2 mm temporal; T1.5, 1.5 mm temporal; T1, 1 mm temporal; F0, foveal thickness; N0.5, 0.5 mm nasal; N1, nasal 1 mm; N1.5, 1.5 mm nasal; nasal N2, 2 mm; N2.5, 2.5 mm nasal; N3, 3 mm nasal.

**Figure 4:**
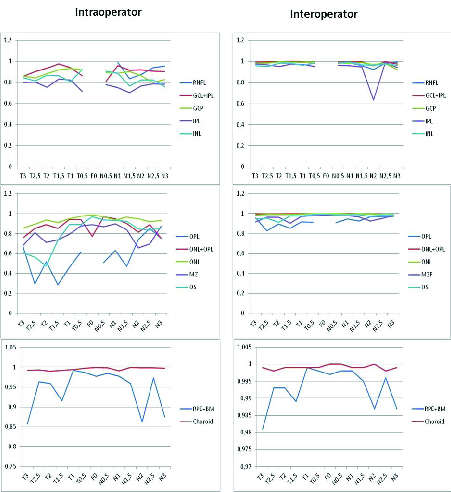
(A) Intra-operator inner layers. (B) Intra-operator outer layers. (C) Intra-operator RPE/MB complex and choroid. (D) Inter-operator inner layers. (E) Inter-operator outer layers. (F) Inter-operator RPE/BM complex and Choroid. T3, 3 mm temporal; T2.5, 2.5 mm temporal; T2, 2 mm temporal; T1.5, 1.5 mm temporal; T1, 1 mm time; F0, foveal thickness; N0.5, 0.5 mm nasal; N1, nasal 1 mm; N1.5, 1.5 mm nasal; nasal N2, 2 mm; N2.5, 2.5 mm nasal; N3, 3 mm nasal.

### SD-OCT

In this work, the SD-OCT (*Cirrus* HD-OCT Version 6.5; *Carl Zeiss Meditec)* scanning protocol included an HD 5-line raster (4,096 A-scans for each of the five B-scans) with an axial resolution of approximately 6 µm. According to the hospital protocol, all HD 5-line raster scans were studied, with a spacing of 0.25 mm and using the enhanced depth imaging^[17]^ mode without image inversion centered at the fovea.

The analysis and quantification of the horizontal B scan passing through the fovea were performed with a 2 × zoomed image. The process began with the identification and marking of the fovea, using the “caliper function.” All the layers' measurements were obtained in six places, with intervals of 500 µm up to temporal (T) 3 mm. The procedure was repeated for the nasal (N) region (3 mm). The description of the various layers (Figure 1) was made based on the international nomenclature for OCT (IN-OCT consensus).^[18]^

### Statistical Analysis

Statistical analysis was performed using SPSS software *(SPSS statistics 22 for Windows; *SPSS Inc., IBM, Somers, NY*).* To compare the age differences, BCVA, and IOP between females and males, Student's *t*-test for independent samples was used after verifying the normality of the sample. A 95% confidence interval and a 5% level of significance were adopted.

The analysis of the repeated measures by the key operator was calculated by the intra-class correlation coefficient (ICC) based on variance. The study measured 12 layers of the retina and the choroid in 40 eyes of 40 participants at 13 locations along the horizontal meridian, from F0, with 169 measurements per participant. In the intra-operator study, these measurements were repeated at three time points (18,720 measurements in a total of 40 eyes). Additionally, with the collaboration of the two trained operators, over 12,480 measurements (6,240 per operator, in an equal number of participants) were made at the same locations.

With the recourse of an adapted ICC, we also studied and compared the measurements performed by the two operators, with the third moment from the key operator (inter-operator variability). In total, the ICC was calculated 312 times (single measures).

ICC values can vary between 0 and 1, which represents a perfect correlation. We defined the values of 0.8–0.9 as good and those > 0.9 as excellent.

##  RESULTS

The study consisted of 40 eyes of 40 participants (30% men and 70% women) with an average age and standard deviation of 76.4 ± 8.3 (range, 55–92 years).

No statistically significant age difference was found between genders: *p* = 0.749 (Student's *t*-test for independent samples: men 75.4 ± 8.1 and women 76.6 ± 8.4).

Regarding BCVA, no difference was observed between genders (*p* = 0.546), with a higher average number of letters in women (76.6 ± 6.5) and a lower average number in men (75.8 ± 4.7). No statistically significant difference was found in terms of IOP between genders (*p* = 0.784, men 15.6 ± 2, women 15.7 ± 2.2).

### Retinal and Choroid Segmentation

Regarding segmentation of the retinal layers and the choroid [Figure 3], the values are presented below. In the inner layers [Figure 3A], the first values were registered at the level of the retinal nerve fiber layer (RNFL), in N1, with an average value of 8 ± 7.2 µm, which gradually increased to the maximum average value of 50 ± 15.7 µm in N3. At the level of the ganglion cell layer with the inner plexiform layer (GCL+IPL complex), the highest value was obtained in N0.5 (86.9 ± 22.0 µm), and the clusters of higher values were registered between the N2 and T2.

Among the outer layers [Figure 3B], the outer plexiform layer (OPL) showed very close values in all measurements. In the outer nuclear layer with an OPL (ONL+OPL complex), we revealed the maximum peak in the F0 (96 ± 32 µm) and the 1500 µm central macula met the highest values in this structure. In the ONL, the maximum peak was 96 ± 32 µm (F0). However, this layer presents a more remarkable centrifuge decrease in the nasal and temporal regions from 500 µm. Most of the myoid zones (MZ) of the photoreceptors' average values are slightly larger than the outer segments (OS) of photoreceptors' values. The exception was found in F0 where this trend was reversed (MZ = 25.9 ± 8.4 µm vs OS = 30.2 ± 13.3 µm).

In relation to the RPE+BM complex, we found most thickness values between T1 (43.2 ± 31.6 µm) and N1 (42.6 ± 33.1 µm). Finally, at the level of the choroid, we found the highest values at the F0 (217.1 ± 108.3 µm). All values are shown in Table 1.

The variability of the repeated measures is presented in Table 2 (summary ICC for a CI of 95% and *p*-value < 0.001) and Table 3 (summary of adapted ICC for a CI of 95% and *p*-value < 0.001). The graphical representation of these values are shown in Figure 4.

### Intra-operator Variability 

For the inner layers and starting of the RNFL, the minimum ICC was obtained in N1.5, with a value of 0.833 (0.736; 0.902). All remaining ICC values were > 0.9, with a maximum value of 0.992 (0.986; 0.995) in N1. In the GCL+IPL complex, the minimum ICC value was 0.860 (0.776; 0.918) at T3. The highest value was 0.974 (0.956; 0.985) at T1.5.

Except for the ICC of 0.800 (0.688; 0.881) at N2.5, in the GCL, all the other values were > 0.826 (0.726; 0.898) at N3. The best ICC values were located between T0.5 and T1.5. The ICC of the IPL measurements varied between 0.703 (0.556; 0.818) at N1.5 µm and 0.831 (0.734; 0.901) at T1.5 µm.

At the level of the inner nuclear layer, the ICC values in most situations were > 0.80. In the OPL, the lowest ICC values were observed in T2.5, with 0.293 (0.096; 0.501) and approximately 0.286 (0.089; 0.495) in T1.5.

In the ONL+OPL complex, except for the extreme locations (T3 or N3), the remaining ICC values were > 0.81. Similarly, the ONL exhibited the lowest ICC value of approximately 0.857 (0.773; 0.917) in T3 and the remaining values were > 0.894 (T2.5).

At the level of the MZ, the lowest values were 0.683 (0.530; 0.805) in T3 and 0.657 (0.497; 0.787) and 0.697 (0.548; 0.814) in N2 and N2.5, respectively. Higher ICC values were found in the proximity of the fovea. Similarly, in the OS, higher values of ICC were measured near the fovea.

At the level of the RPE+BM complex, the lowest values were observed at extreme positions such as T3 (0.857) or N3 (0.875). All remaining ICC values were > 0.91. In relation to the choroid, all ICC values obtained were > 0.990 (0.984; 0.995) registered in T2.

### Inter-operator Variability 

Regarding the values obtained for the inter-operator variability, the lowest value obtained at the level of the RNFL was 0.923 (0.873; 0.956) in N2. In the GCL+IPL complex, the lowest value measured was 0.989 (0.981; 0.994) at T0.5, and the remaining values were higher. In the GCL, the lowest value was in an eccentric location, with approximately 0.924 (0.875; 0.957) in N3. At the level of the IPL, except for N2, all the remaining values obtained were > 0.949 (0.915; 0.971) registered in N1.5.

In the ONL+OPL complex, the values obtained were > 0.993 (0.988; 0.996) registered in N2.5. In the ONL, we found two minimum records of 0.985 (0.975; 0.992) and 0.988 (0.980; 0.994) in T3 and T2.5, respectively.

In the MZ, the lowest values were found in the temporal region with approximately 0.903 (0.842; 0.944) in T1.5 and 0.918 (0.865; 0.953) in T3.

At the level of the OS, the highest values were found in the nasal region. In this location, the lowest value was 0.972 (0.953; 0.984) in N2. The value obtained in the fovea was 0.995 (0.991; 0.997).

At the RPE+BM complex, the lowest values found in the peripheral locations were 0.981 (0.968; 0.989) in the temporal region and 0.987 (0.978; 0.993) in the nasal region. The fovea had a value of 0.997 (0.996; 0.999).

Regarding the choroid, the obtained values were between a minimum of 0.998 (0.997; 0.999) in T2.5 and 0.998 (0.996; 0.999) in N2.5, and a maximum of 1 (1; 1) at F0.

##  DISCUSSION

The evolution and widespread use of SD-OCT in clinical practice has revolutionized the detection and follow-up of retinal pathologies.[1–3] Despite the success of this clinical utilization due to its reproducibility,^[2,5]^ several researchers have studied the validity and reproducibility of its quantification and retinal segmentation.^[1,7,8,9,19]^ Even with a more modern approach, like DL methods, accurate layer segmentation remains crucial for appropriate clinical interpretation, especially in the presence of retinal structural changes.^[13,14]^

### Segmentation

According to RNFL segmentation, as expected, we found the highest values in N3 (50 ± 15.7 µm). At the level of the GCL, GCL+OPL complex, IPL, and ONL layers, the highest values were found in the central 3 mm. As already reported by Curcio et al^[3]^ and Anger et al, slight differences were noticed between the nasal and temporal anatomy. The largest average values of these layers were found in the nasal region. We observed values with the greatest variation in different positions in the OPL+ONL complex. The MZ and OS layers represented areas with little local variation. With higher thickness values and more local variations than the two previous segmentations, the RPE+BM showed a maximum thickness in the fovea (45.4 ± 25.6 µm). Considering the choroid, we found the same trend with the highest values located in the central 3,000 µm. In this structure, we found a centrifugal decrease in the maximum values of the fovea (217.1 ± 108.3 µm), particularly in the nasal region.^[20]^ In summary, except for the RNFL, the regions of greater thickness were located in the central 3 mm of the macula. This central macular area seems to have a condition conducive for assessment and monitoring. Previous reports showed a special interest in the local central area (between the N1.5 and T1.5) where small pathological changes could be easily detected.^[4]^ These seem to be regions garnering the greatest interest in studies[3–4] and may also be of great clinical relevance.

### Variability of the Data

Globally, all the layers showed good levels of reproducibility and repeatability.^[7]^ Regarding intra-operator variability, the best ICC obtained was found at the locations of the greatest thickness. In this sense, the area of greatest thickness at the RNFL (N3) presented an ICC of 0.953 (0.922; 0.973). Curiously, we observed that the reproducibility of data was better when two layers were studied together, like the GCL+IPL complex.

The IPL and OPL segmentations exhibited the lowest ICC values, similar to previous studies.^[2,5]^ This may be due to the smaller thickness when compared to the remaining layers, but also due to the reflectivity of these layers, which decreases the reproducibility. The complexity of the structure itself and its anatomy^[15]^ may also be another factor that influenced the obtained values, reinforcing the need for special attention toward the correct measurement of the frame (caliper position).

Among the remaining outer layers, the ICC values were close to 0.90 in the region of the fovea, as in other studies.^[2,19,21]^

The RPE+BM complex was verified as the thinnest structure with larger ICC values. The choroid presented excellent ICC values of > 0.99.

In conclusion, automatic quantification is faster, but the accuracy of manual quantification continues to be higher than that of automatic measurements.^[19,22]^ The excellent values of reproducibility of manual quantification with an ICC of 0.92 were described.^[23]^ In this work, except for the 0.826 (0.727; 0.898) obtained in the OPL (T2.5) and 0.634 (0.469; 0.771) obtained in the IPL (N2), all correlation values were > 0.92, and, in most cases, > 0.98.

Regarding manual quantification, it has been proven that, despite the subjectivity introduced by human involvement,^[2]^ very high ICC values can be achieved with training and by following the standard protocol. This aspect was well-represented by the excellent set of correlation values obtained in our inter- and intra-operator analyses.

##  Limitations

Manual segmentation can be considered a limitation. Nowadays, most studies in this area focus on healthy participants using automatic algorithms with good reliability.^[1,5,9,15,24,25]^ However, in the presence of retinal pathology, manual segmentation or manual correction is repeatedly mentioned as being essential for decreasing measurement variability and improving reproducibility.^[10]^

Despite the anatomical difficulties, such as the OPL,^[2,5]^ we highlight three points in the segmentation and measurements: (a) the values found in the retinal and choroidal segmentation, in conjunction with the ICC values obtained for each of the 13 points, can help to build an optimized data protocol by SD-OCT for the assessment and monitoring of retinal pathology and other small changes as described in AMD and/or aging;^[26,27]^ (b) regardless of the layer thickness, their boundaries and good reflectivity seem to be the most important aspects to obtain accurate measurements; (c) quantification of the layers in complex forms (such as GCL+IPL) allows more reproducible measurements even with low reflectivity boundaries.

##  Financial Support and Sponsorship

Nil.

##  Conflicts of Interest

None of the authors has any conflicting financial interests related to this study.
